# Prediction of Electron Beam Welding Penetration Depth Using Machine Learning-Enhanced Computational Fluid Dynamics Modelling

**DOI:** 10.3390/s23218687

**Published:** 2023-10-24

**Authors:** Yi Yin, Yingtao Tian, Jialuo Ding, Tim Mitchell, Jian Qin

**Affiliations:** 1 Department of Engineering, Lancaster University, Bailrigg, Lancaster LA1 4YW, UKy.tian12@lancaster.ac.uk (Y.T.); 2 WAAM3D Ltd., 7 Thornton Chase, Milton Keynes MK14 6FD, UK; jialuo@waam3d.com; 3 TWI Ltd., Granta Park, Great Abington, Cambridge CB21 6AL, UK; tim.mitchell@twi.co.uk; 4 Welding and Additive Manufacturing Centre, School of Aerospace, Transport and Manufacturing, Cranfield University, Bedfordshire MK40 3AA, UK

**Keywords:** electron beam welding, computational fluid dynamics modelling, machine learning, artificial neural networks, penetration depth prediction, beam characterisation

## Abstract

The necessity for precise prediction of penetration depth in the context of electron beam welding (EBW) cannot be overstated. Traditional statistical methodologies, including regression analysis and neural networks, often necessitate a considerable investment of both time and financial resources to produce results that meet acceptable standards. To address these challenges, this study introduces a novel approach for predicting EBW penetration depth that synergistically combines computational fluid dynamics (CFD) modelling with artificial neural networks (ANN). The CFD modelling technique was proven to be highly effective, yielding predictions with an average absolute percentage deviation of around 8%. This level of accuracy is consistent across a linear electron beam (EB) power range spanning from 86 J/mm to 324 J/mm. One of the most compelling advantages of this integrated approach is its efficiency. By leveraging the capabilities of CFD and ANN, the need for extensive and costly preliminary testing is effectively eliminated, thereby reducing both the time and financial outlay typically associated with such predictive modelling. Furthermore, the versatility of this approach is demonstrated by its adaptability to other types of EB machines, made possible through the application of the beam characterisation method outlined in the research. With the implementation of the models introduced in this study, practitioners can exert effective control over the quality of EBW welds. This is achieved by fine-tuning key variables, including but not limited to the beam power, beam radius, and the speed of travel during the welding process.

## 1. Introduction

An electron beam (EB) is an ideal heat source for metal joining in various scenarios, as its large aspect ratio and the distortion accumulation caused by electron beam welding (EBW) can be significantly reduced compared with plenty of traditional joining methods [1]. A notable molten pool is often generated during EBW, and in fully penetrated welding, there is less solid support under the hot liquid metal, which potentially leads to collapse of the weld joint. Partially penetrated welds are an effective approach to prevent collapse caused by liquid metal gravity, and provide a suitable fusion zone pattern. The post process of partially penetrated EBW largely depends on the weld penetration depth, and the penetration depth can be much different with varied beam parameters or welding settings such as the beam current, accelerating voltage, travelling speed, focal spot size, etc. [2]. For EBW operators, accurately estimating the penetration depth based solely on these figures and settings is challenging, especially when welding a new material for the first time. Therefore, there is a direct demand of a developing reliable EBW penetration depth prediction approach.

Numerous studies have been dedicated to using computational fluid dynamics (CFD) models for replicating the physical phenomena of EBW and guiding manufacturing processes. Taking into account the purpose of the models and complexity of boundary conditions, both 2D versions and 3D versions are frequently used, all of which exhibit the potential to predict penetration depth. While the 2D models can provide basic simulation results, they are particularly useful in specific EBW scenarios.

Liu and He [3] introduced a keyhole tracking method, and applied it to simulate aluminium alloy spot weld pool dynamics. The model successfully reproduced the formation of vapour cavities caused by keyhole collapse. However, it was limited to spot welds without a moving heat source, making it difficult to verify its results in traditional butt weld situations. To address this limitation, they simplified the model to a 2D version in 2017, and also found that keyhole collapse was the main reason for penetration stopping [3].

In another study, Tomashchuk et al. [4] developed a 2D CFD model to simulate the propagation of dissimilar liquid metals during EBW of pure copper with AISI 316 austenitic stainless steel. This model successfully predicted different types of molten pool morphologies, and provided calculated copper/steel fractions in the melted zone. The study also revealed that residual stresses decreased when copper filler was added during the Ti-15-3 alloy to 304 stainless steel EB welding process. However, due to the lack of calculation in one flow direction, predicting penetration depth efficiently is challenging with 2D models when compared to 3D models.

The 3D CFD models in previous studies open up new possibilities for predicting EBW penetration depths. For instance, the 3D CFD method has been successfully applied to various materials, as demonstrated by Borrmann et al. [5], who used it to simulate EB-welded TRIP/TWIP steels with different thermophysical properties. Liang et al., employed a CFD–FEM model to simulate the residual stress in EB-welded Ti-6-Al-4V alloy caused by spiking defects [6], and Chen et al. utilised CFD simulations to study porosity defects during the EBW process of the 2A12 aluminium alloy [7,8]. Therefore, with proper material properties, the welding process of most metal types can be simulated using 3D CFD models.

Moreover, 3D CFD models have successfully simulated the generation of keyholes inside the molten pool and the physical phenomena during the EBW process. For instance, B. Huang’s 3D CFD model based on the level set (LS) method effectively simulated weld pool dynamics and keyhole generation [9]. The simulation revealed a 1–2 kHz keyhole oscillation, with certain high welding speeds stabilising the keyhole and flow speeds inside the molten pool reaching 5 m/s. Other studies have also employed similar LS methods to simulate the EBW process of Ti-6Al-4V alloy [10,11]. One of these studies focused on keyhole and molten pool dynamics during scanning electron beam welding, finding that high-frequency beam scanning resulted in better uniformity of the keyhole and fewer defects compared to low-frequency welds. The other study investigated vapour plume dynamics inside the EBW keyhole, reporting a maximum velocity of 1500 m/s based on simulated results.

Furthermore, 3D CFD modelling successfully replicated various fusion zone patterns of EBW. Wang et al. [12] used a combined point-linear heat source to simulate the temperature field of an electron beam-welded titanium alloy, successfully replicating various fusion zone patterns such as the nail pattern, bell pattern, funnel pattern, and chock pattern.

Additionally, 3D CFD simulations have demonstrated their potential in guiding weld parameter selection for real EBW production. D. Trushnikov and G. Permyakov introduced a 3D CFD model in 2017 to investigate the influence of focus position on the molten pool dynamics of steel welded by oscillated EBW [13]. The results, both experimental and simulated, showed that the amplitude of convection caused by the Marangoni effect increased when the beam focusing position moved downward. Yin et al. [14] utilised the CFD method to simulate the weld bead shape of an electron beam-welded pure niobium plate, aiming to obtain dependable electron beam oscillation parameters for use in manufacturing superconductivity cavities.

The earlier study highlighted that CFD modelling holds promise in predicting penetration depth and even other dimensions of the molten pool. Moreover, the 3D models can offer a more dependable prediction, as they can incorporate a greater amount of boundary information. However, the CFD models mentioned above are mostly focused on quality variations in the EBW process rather than on providing specific quantities to guide EBW parameter selections. Due to the complexity of the solid–liquid–gas transformation during keyhole generation, there is a lack of reliable CFD models capable of predicting the penetration depths for EBW processes that last a few seconds.

On the other hand, obtaining a prediction result from CFD modelling frequently takes hours or even days. Additionally, adept skills in model setup are crucial for achieving a highly dependable solution. These factors render the application of CFD prediction challenging in numerous industrial scenarios, especially those necessitating real-time solutions in production. As a result, for predicting EBW penetration depth, the prevailing approach remains reliant on statistical tools like regression fitting and artificial neural networks (ANN).

For example, Dey et al. [15] in 2008 utilised a backpropagation neural network (BPNN) and a genetic algorithm-tuned neural network (GANN) to achieve the EB weld bead profile predictions of stainless steel 304. Moreover, they applied a similar methodology to predict the EB weld bead profile of other materials, including aluminium alloy [16,17,18] and reactive material zircaloy-4 [19]. According to the studies conducted by Shen et al. in 2009 [20], they also utilised BPNN to predict weld bead dimensions for 1cr18ni9ti stainless steel. The maximum absolute value error for the predicted penetration depth was 6.6%.

Such methods offer the advantage of being straightforward to implement, and generally provide reliable results when appropriate tests are conducted and prediction parameters are within a specified range. However, when applying these methods to a different material, statistical approaches require extensive training data, which is typically expensive and time-consuming.

Several studies have also used empirical equations to predict the penetration depth of EBW. However, some of these equations are not available before conducting the weld [21], while others are challenging to implement in real industrial production due to their unclear beam radius characterisation method [22,23].

Combining numerical simulations with artificial intelligence algorithms has been demonstrated as an efficient approach for addressing numerous industrial challenges. These challenges often involve either the unavailability of extensive experimental data or complexities influenced by various factors. Examples include predicting the size of flammable vapour clouds caused by liquid hydrogen release [24], solving problems related to natural convection and conjugate heat transfer [25], predicting solid particle erosion in the oil and gas industry [26], and enhancing the design of centrifugal pumps [27], etc. Based on these studies, the integrated model has shown promising potential in guiding manufacturing processes, explaining physical rules, and enhancing industrial safety.

In this research, a machine learning (ML) enhanced CFD model is introduced that accurately and efficiently predicts the penetration depth during electron beam welding. Several improvements have been made in comparison to previous studies:

- Characterisation of electron beams: The electron beams are characterised using the method described in [2], allowing the beam radius in both the welding and cross-sectional directions to be identified. As a result, the beam characteristics can be easily incorporated into the CFD model as inputs, following a standard protocol.
- Simulation of a 2-s welding process: The CFD model has been adjusted to simulate a 2-s welding process, incorporating an efficient and easily understandable heat generation algorithm.
- Applications for neural network training: the CFD model is also suitable for training a neural network model. This allows for rapid and reliable penetration depth predictions in real industrial environments.

The predicted data are compared with experimental data, and mild steel S275JR was chosen for the partially penetrated experiments to validate the feasibility of this method. Through these measures, this approach can provide accurate penetration depth predictions, can be adapted to different materials, eliminates the need for expensive and time-consuming trial and error tests, and can be integrated into an ML model to meet the requirements of Industrial 4.0.

## 2. Methodology

**Figure 1:**
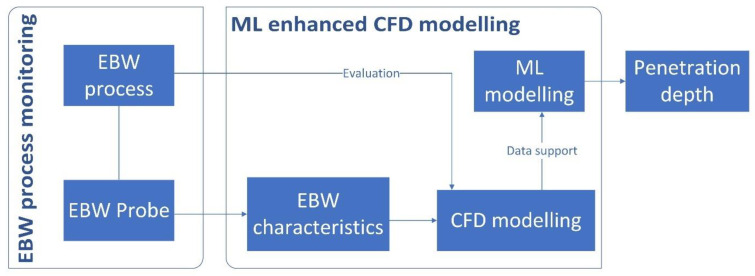
Schematic diagram of ML enhanced CFD modelling for EBW penetration depth prediction.

The process of ML-enhanced CFD modelling is illustrated in Figure 1. Initially, EBW tests are carried out, during which the characteristics of the electron beam are detected. Subsequently, the CFD model designed to replicate the EBW process is established. The data collected in the initial phase are employed to validate the accuracy of the CFD model. Once the simulated penetration depth from the CFD aligns with the requirements of prediction, the model can be utilised to generate virtual data that support the ML model. Then, the trained ML model is employed to forecast the penetration depth in real experiments and assess the accuracy of these predictions.

## 3. EBW Process Monitoring

The welding experiments were conducted using an EB machine developed by Cambridge Vacuum Engineering (serial no. CVE 661), with a maximum power of 4 kW and a maximum accelerating voltage of 60 kV. The experiments were carried out under a vacuum level of 10^−3^ mbar. The experimental conditions were varied within the following ranges: 40–60 kV for the accelerating voltage, 25–45 mA for the beam current, and a welding speed of 500–700 mm/min. The EB linear power was controlled in the range of 86 J/mm to 324 J/mm. The focusing currents were adjusted between 277 mA and 366 mA, resulting in three different focal conditions: over-focus, sharp-focus, and under-focus. Over-focus refers to the beam focal position above the sample surface, sharp-focus at the surface level, and under-focus below the surface level. The beam radii were characterised using the 4-slit probe developed by The Welding Institute (TWI), UK [28]. The working distance, measured from the chamber roof to the workpiece surface, was 157 mm.

**Figure 2:**
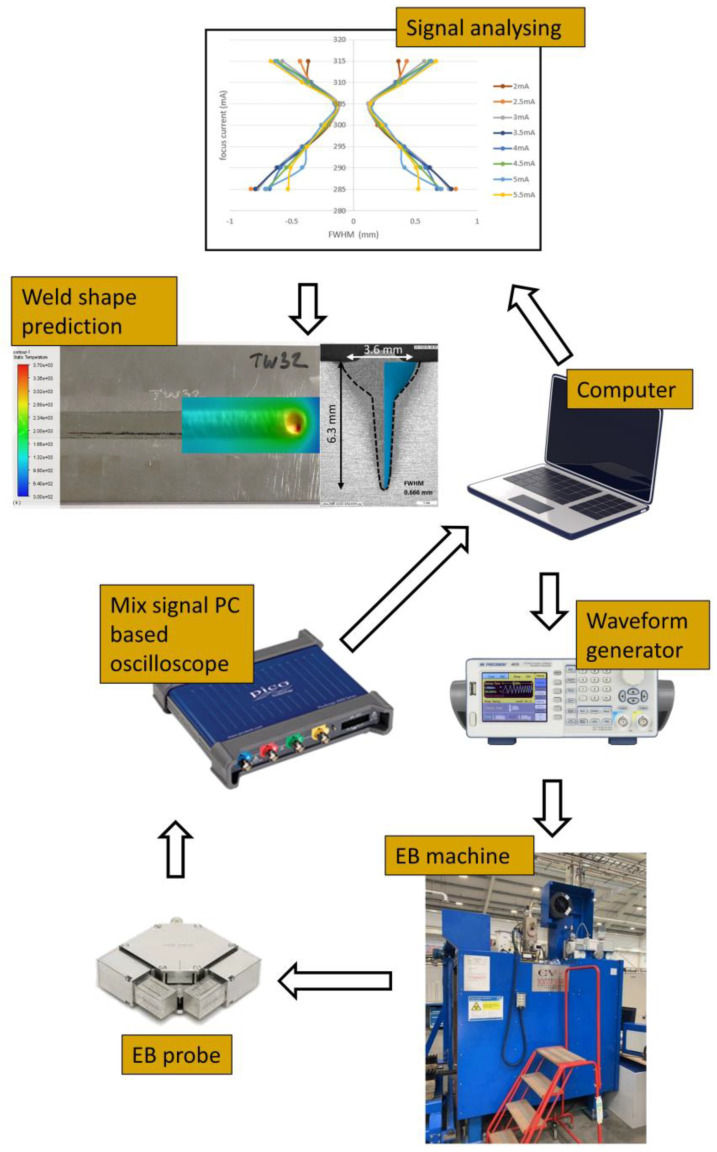
Schematic of EB welds on a S275JR mild steel plate.

**Figure 3:**
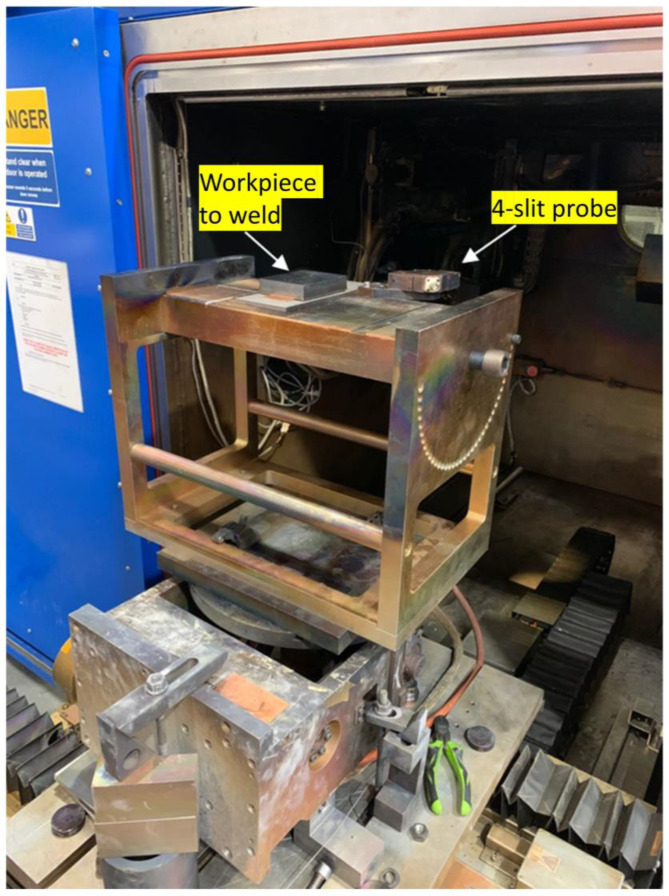
An example of placement of a workpiece to weld and the 4-slit probe.

A sketch of the beam probing system setup is illustrated in Figure 2. The system comprises an electron beam (EB) machine, an EB probe, a PC-based oscilloscope, a computer, and a waveform generator. The EB probe was installed inside the vacuum chamber of the EB machine and connected to an oscilloscope outside the chamber via the chamber interface. The probe’s working surface with slits was positioned under the electron beam gun and perpendicular to the electron beam path. To ensure precise beam parameters, the probe’s working surface was aligned with the surface of the workpiece to be welded. The installed probe is shown in Figure 3.

**Figure 4:**
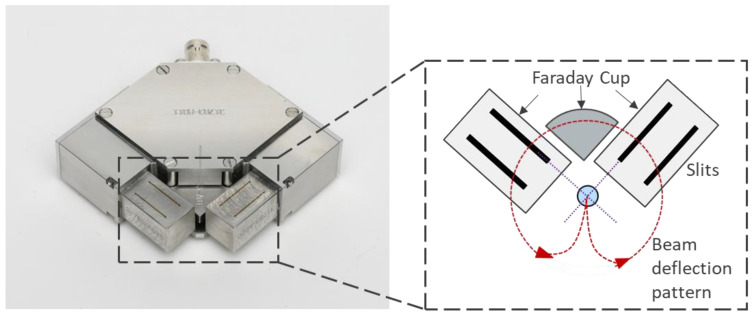
Modified 4-slit probe developed by TWI Ltd., Cambridge, UK.

**Figure 5:**
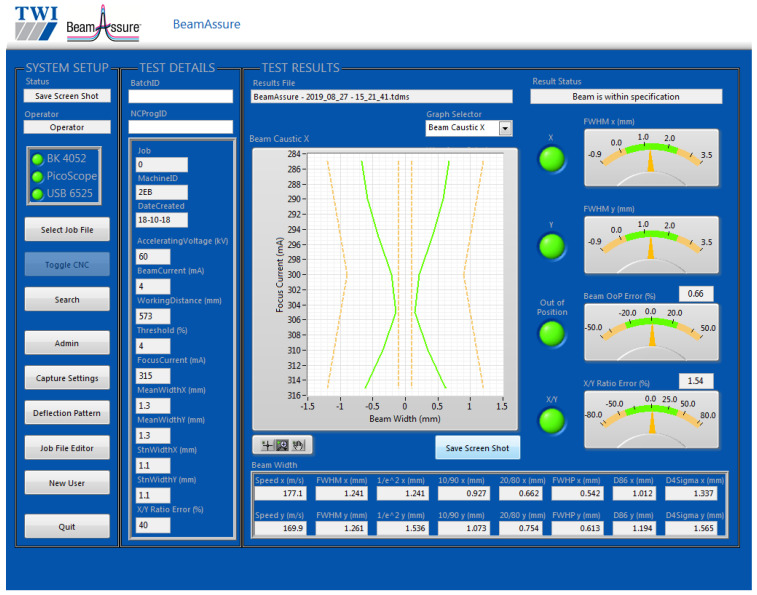
User interface of BeamAssure^TM^ electron beam characterising system. Yellow dashed lines represent the predefined beam radii range, while the green lines represent the actually detected beam radii.

The electron beam is rapidly deflected to scan across the slits of the EB probe for beam characterisation, as depicted in Figure 4. A specific deflection pattern can be designed on the computer and converted into a series of signals to control the deflection coils in the EB machine through a waveform generator. Electrons passing through slits in multiple directions can be converted into a voltage signal by a Faraday cup positioned beneath and subsequently captured by an oscilloscope. A Faraday cup is an instrument used to measure electric charge or the current of charged particles, typically in electron beams. And the voltage signal, which refers to the beam width information, is visualised on the computer screen by the BeamAssure^TM^ (TWI Ltd., Cambridge, UK) electron beam characterising system shown in Figure 5 [28]. In Figure 5, the ‘System Setup’ and ‘Test Details’ boxes record relevant beam parameters that link beam parameter information with beam radius data. The ‘Test Results’ box displays detected beam radii in the x or y directions. The ‘Beam Caustic’ graph illustrates beam radius changes with varying beam-focusing currents, allowing the determination of the focusing status (‘over-focus’, ‘sharp-focus’, and ‘under-focus’). In the ‘Beam Width’ box, values for differently defined beam radii can be found. The system can issue a warning signal if any detected value exceeds the preset limit. These beam probing data can then be further analysed and applied in the CFD model. Once the beam probing process is complete, the welding process can begin.

S275JR mild steel was used as the substrate material, with dimensions of 100 × 75 × 20 mm. More details of the experiment design, beam probing, sample treatment and measurement data have been published in [2]. Additionally, Table 1 presents the measured depths obtained under various parameters.

## 4. ML-Enhanced CFD Modelling

### 4.1. CFD Model Setup

**Figure 6:**
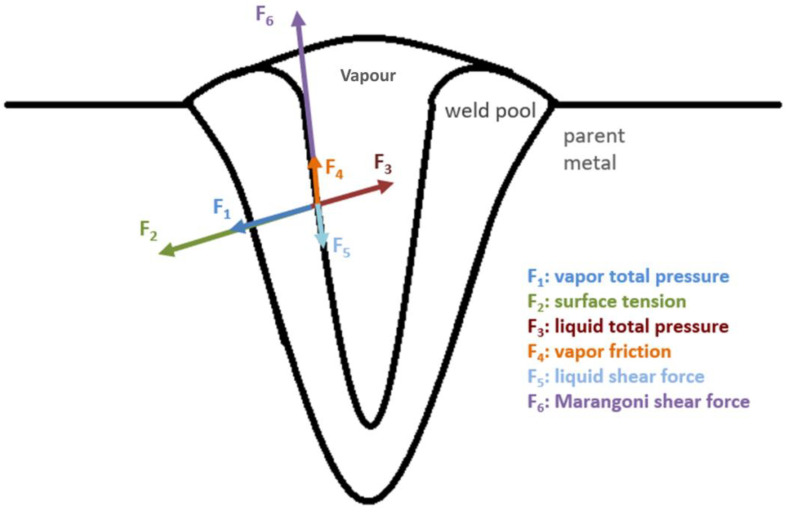
Primary forces operating at the molten pool interface during EBW.

Figure 6 illustrates the primary forces acting on the surface of the molten pool during deep-penetrated EBW. When electrons impact the metal surface to be welded, energy is released, causing the parent metal to melt and form a molten pool. At the interface of the molten pool, various forces come into play, including vapour pressure, liquid pressure, vapour friction, liquid shear force, and Marangoni shear force. These forces collectively contribute to the formation of a deep and narrow keyhole. To predict the weld depth, these phenomena are replicated and simulated within the CFD model.

**Figure 7:**
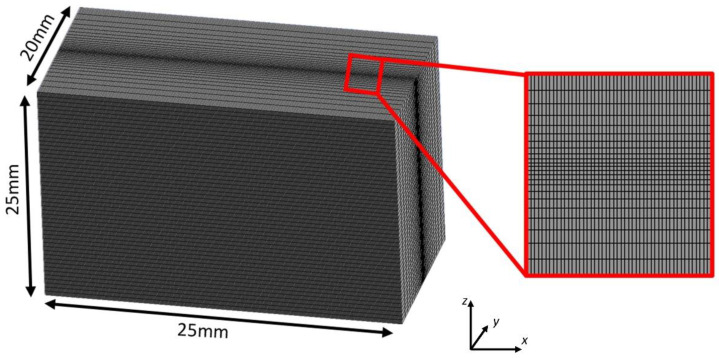
Model dimensions setup and mesh design.

Computational fluid dynamics modelling was performed using ANSYS Fluent 2020R2, incorporating user-defined functions (UDF) written in C++. The simulation was conducted on the high-end computing (HEC) infrastructure at Lancaster University, utilising a 6-core Intel 10th-generation CPU for each model. The mesh design of the model is presented in Figure 7. Initially, elements in the bottom 20 mm along the z-direction were set as the metal solid phase, while the remainder was set as the air gas phase. Both the solid and gas phases are transferable, and were included in the calculation domain. The boundaries attached to the solid phase, located in the bottom 20 mm along the z-direction, were set as thermal insulation walls, while the remaining boundaries were designated as pressure outlets. Notably, the mesh size gradually decreased from the outer elements towards the inner elements adjacent to the weld seam. A mesh independence test was conducted by varying the minimum element size from 1 mm to 0.1 mm. It was observed that when the meshing size fell below 0.2 mm, the average simulated penetration depth reached a relatively constant value after 0.8 s of weld time. Consequently, the model utilised 453,900 hexahedron elements with a minimum edge size of 0.15 mm to ensure accuracy. To optimise prediction efficiency and mitigate non-convergence issues, a fixed simulation time step of 0.5 ms was employed. The simulation should continue for approximately 1 s of welding time after the simulation reaches a relatively constant depth. This extended duration is necessary to capture depth measurements at different time intervals for calculating the average depth and standard deviation. As a result, the total simulated weld time amounts to 2 s. During the simulation, several assumptions and simplifications were made:

- The molten pool was assumed to exhibit laminar flow, be incompressible, and behave as a Newtonian fluid. Based on previous simulation results, applying laminar flow and incompressible Newtonian fluid can successfully reproduce the EBW joining process [29]. The simulation complexity can be reduced without much impact on the simulation results.
- The electron beam power intensity was assumed to follow an ideal Gaussian distribution. This assumption may lead to discrepancies in CFD predictions, since the actual beam power may not strictly follow a Gaussian shape. This simplification, nonetheless, facilitates the determination of power distribution based on the beam radius.

The material properties for S275JR used in the CFD model are provided in Table 2.

The essential settings of the Fluent model can be found in Table 3.

To reproduce the morphology after molten pool solidification, it is essential to set the interface type as “Sharp”. For the mushy zone parameter, a value of 4,000,000 should be selected, as setting it too low could result in undesired movement of the solid phase, while a high value may lead to convergence issues. The remaining settings were determined either by referring to previous EBW models or using default values.

The VOF (volume of fluid) interface tracking method was utilised, taking into account both computing efficiency and model convergence. The free surface was determined based on the following equation:

(1) $$\frac{\partial F}{\partial t}+\frac{\partial F}{\partial x}+\frac{\partial F}{\partial y}+\frac{\partial F}{\partial z}=0$$

In the equation, F represents the volume fraction of the metal phase. The interface cells are tracked when the value of F lies between 0 and 1.

The equations governing mass, momentum, and energy conservation are described below [8]:

(2) $$\frac{\partial \rho }{\partial t}+\frac{\partial (\rho u)}{\partial x}+\frac{\partial (\rho v)}{\partial y}+\frac{\partial (\rho w)}{\partial z}=0$$

where u, v, and w denote the velocity components in the x, y, and z directions, respectively, and ρ represents the density.

(3) $$\frac{\partial (\rho u)}{\partial t}+\frac{\partial (\rho uu)}{\partial x}+\frac{\partial (\rho uv)}{\partial y}+\frac{\partial (\rho uw)}{\partial z}=-\frac{\partial p}{\partial x}+\frac{\partial }{\partial x}\left(\mu \frac{\partial u}{\partial x}\right)+\frac{\partial }{\partial y}\left(\mu \frac{\partial u}{\partial y}\right)+\frac{\partial }{\partial z}\left(\mu \frac{\partial u}{\partial z}\right)+P_{rx}+F_{\sigma x}$$

(4) $$\frac{\partial (\rho v)}{\partial t}+\frac{\partial (\rho vu)}{\partial x}+\frac{\partial (\rho vv)}{\partial y}+\frac{\partial (\rho vw)}{\partial z}=-\frac{\partial p}{\partial y}+\frac{\partial }{\partial x}\left(\mu \frac{\partial v}{\partial x}\right)+\frac{\partial }{\partial y}\left(\mu \frac{\partial v}{\partial y}\right)+\frac{\partial }{\partial z}\left(\mu \frac{\partial v}{\partial z}\right)+P_{ry}+F_{\sigma y}$$

(5) $$\frac{\partial (\rho w)}{\partial t}+\frac{\partial (\rho wu)}{\partial x}+\frac{\partial (\rho wv)}{\partial y}+\frac{\partial (\rho ww)}{\partial z}=-\frac{\partial p}{\partial z}+\frac{\partial }{\partial x}\left(\mu \frac{\partial w}{\partial x}\right)+\frac{\partial }{\partial y}\left(\mu \frac{\partial w}{\partial y}\right)+\frac{\partial }{\partial z}\left(\mu \frac{\partial w}{\partial z}\right)+P_{rz}+F_{\sigma z}+F_{b}-G$$

where p denotes pressure, μ represents dynamic viscosity, $P_{r}$ stands for recoil pressure, $F_{\sigma }$ represents surface tension, $F_{b}$ denotes buoyancy force, and G represents gravity. The subscripts x, y, and z refer to vector components in the *x*, *y*, and *z* directions, respectively.

(6) $$\frac{\partial (\rho H)}{\partial t}+\frac{\partial (\rho uH)}{\partial x}+\frac{\partial (\rho vH)}{\partial y}+\frac{\partial (\rho wH)}{\partial z}=\frac{\partial }{\partial x}\left(k\frac{\partial T}{\partial x}\right)+\frac{\partial }{\partial y}\left(k\frac{\partial T}{\partial y}\right)+\frac{\partial }{\partial z}\left(k\frac{\partial T}{\partial z}\right)+Q_{in}-Q_{rad}-Q_{evap}$$

(7) $$H=h_{ref}+\int_{T_{ref}}^{T}C_{p}dT+\beta ∆H_{v}$$

(8) $$\beta =\left\{\begin{array}{c} 0\\ \frac{T-T_{s}}{T_{l}-T_{s}}\\ 1 \end{array}\right.\;\begin{array}{c} T<T_{s}\\ \;T_{s}<T<T_{l}\\ T>T_{l} \end{array}\;$$

where H denotes enthalpy, k represents thermal conductivity, T stands for temperature, $Q_{in}$ represents energy input, $Q_{rad}$ denotes radiation heat dissipation, $Q_{evap}$ represents vapourisation heat dissipation, $h_{ref}$ stands for reference enthalpy, $C_{p}$ represents specific heat capacity, $T_{ref}$ denotes reference temperature, $∆H_{v}$ stands for latent heat of fusion, and $T_{s}$ and $T_{l}$ represent the solidus temperature and liquidus temperature, respectively.

**Figure 8:**
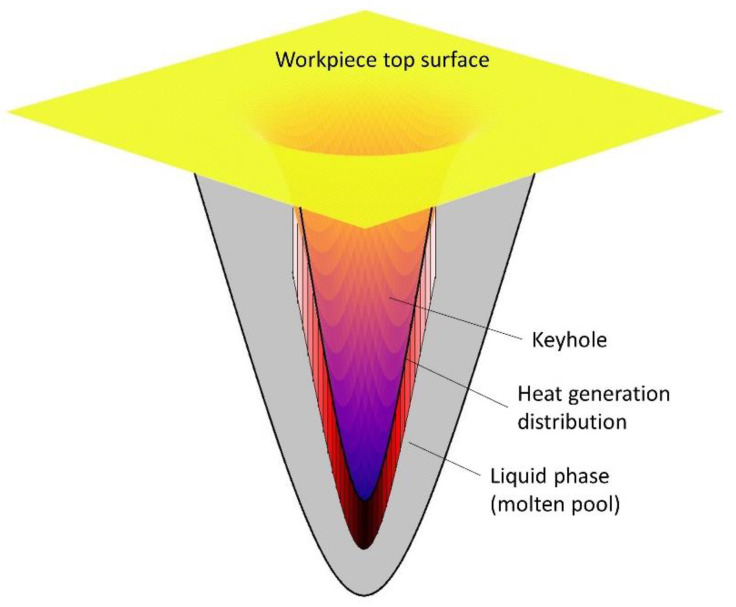
Sketch of heat source generation, with darker red representing higher energy density and vice versa.

In this model, a free-surface tracking Gaussian heat source is utilised, as depicted in Figure 8. The heat source is generated at the metal elements beneath the free surface. The free surface is determined by Equation (1) and follows the power distribution defined by Equation (12). To be more specific, the model scans each element from top to bottom, one by one. During each time step, the element is assigned to either the solid/liquid phase or the gas phase. If an element belongs to the solid/liquid phase and there are not enough solid/liquid phase elements above it, heat will be generated at this element, as per Equation (12). Conversely, no heat will be generated at that element if sufficient solid/liquid phase elements are present above it. This approach allows us to illustrate the heat generation under different conditions; for instance, when the keyhole is insignificant, the heat generated is shallow. Conversely, when the keyhole is deep, the primary heat is generated in a deep area. Lastly, when the keyhole fully penetrates through the workpiece, no energy is generated at the elements of the penetrated column.

The total energy input can be expressed using Equation (9):

(9) $$Q_{in}=U\cdot I\cdot \eta$$

where $Q_{in}$ represents the total input power of the heat source, U is the accelerating voltage, I is the beam current, and η is the assumed efficiency, accounting for convection and evaporation heat loss.

According to [33], η is calculated via the following:

(10) $$\eta =0.6+0.3\left(\frac{z_{w}-z}{0.01}\right)\;for\;0<z_{w}-z\leq 0.01$$

(11) $${\eta =0.9\;\;for\;z}_{w}-z>0.01$$

where $z_{w}$ is the *z* coordinate of the upper surface of the solid phase.

The heat input distribution is cited from [34], and can be expressed as follows:

(12) $$q\left(x,y\right)=\frac{3Q_{in}e^{3}}{\pi \left(e^{3}-1\right)}\times \frac{1}{\left(z_{e}-z_{i}\right)r_{x}r_{y}}\times exp\left(-3\left(\frac{x^{2}}{r_{x}^{2}}+\frac{y^{2}}{r_{y}^{2}}\right)\right)$$

where e is Euler’s number, $z_{e}$ and $z_{i}$ represent the *z*-coordinates at the top and bottom planes of the heat source, respectively. $z_{e}$ is determined using the VOF function, while $z_{e}-z_{i}$ is fixed at 1 mm in this model. $r_{x}$ and $r_{y}$ are the Gaussian distribution radii, referring to the beam radii (1/e^2^ widths) detected by the 4-slit probe mentioned in [2].

In this model, the recoil pressure, hydrostatic pressure and surface tension are considered as driving forces acting at the interface of the liquid metal. The resultant force in the direction normal to the keyhole wall can be expressed as follows:

(13) $$F_{nor}=F_{r}+F_{h}+F_{\gamma }$$

where $F_{r}$, $F_{h}$, and $F_{\gamma }$ represent the forces caused by the recoil pressure, hydrostatic pressure, and surface tension, respectively. In the tangential direction, the resultant force can be expressed by the following:

(14) $$F_{tan}=F_{M}+F_{s}$$

where $F_{M}$ and $F_{s}$ represent the Marangoni shear force and flow shear force, respectively. In this study, the recoil pressure, $P_{r}$, is based on [8] and can be expressed as follows:

(15) $$P_{r}=0.54P_{0}exp\left(L_{v}\frac{T-T_{b}}{RTT_{b}}\right)$$

where $P_{0}$, $L_{v}$, $T_{b}$, and R represent the ambient pressure, latent heat of evaporation, boiling point of the metal, and universal gas constant, respectively.

Since convection and evaporation heat losses have been accounted for in the assumed heat input efficiency η, the thermal boundary of the metal interface can be expressed as follows:

(16) $$k\frac{\partial T}{\partial \vec{n}}=-\xi \psi \left(T^{4}-T_{0}^{4}\right)$$

where *T*_0_ denotes the ambient temperature, while ξ and ψ represent the surface radiation emissivity and the Stefan–Boltzmann constant, respectively.

The thermal insulation wall conditions are governed by the following:

(17) $$k\frac{\partial T}{\partial \vec{n}}=0$$

**Figure 9:**
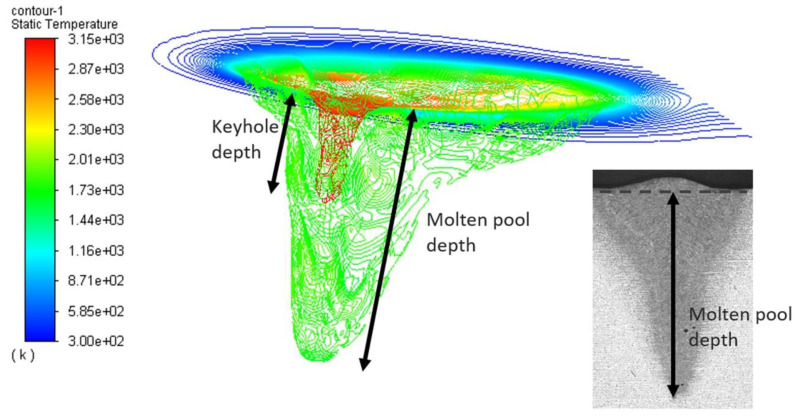
An example of molten pool penetration depth prediction using the CFD method.

Figure 9 illustrates a moment of the simulated molten pool results. The red contour line represents the keyhole profile, while the green contour line indicates the molten pool profile. In this study, the molten pool depth simulated by CFD is represented by the green contour. To calculate the average depth and standard deviation, the simulation runs until depth values can be collected at specific welding intervals: 1.2 s (Depth 1), 1.4 s (Depth 2), 1.6 s (Depth 3), 1.8 s (Depth 4), and 2.0 s (Depth 5). Considering the mesh size settings, the precision of the simulated depth is 0.25 mm. The depth of the liquid phase, standing from the molten pool, was measured using the software ImageJ 1.53e by means of a macro.

### 4.2. ML Model Setup

**Figure 10:**
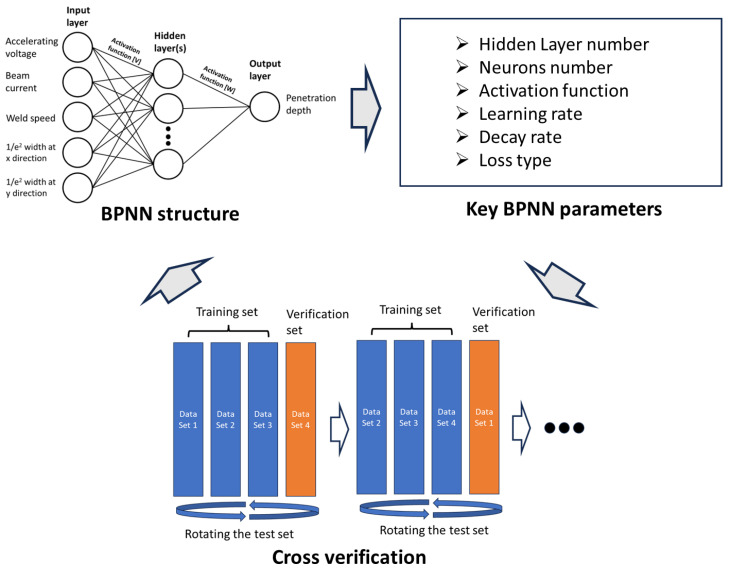
Procedure to determine BPNN structure and parameters.

Utilising the paired data generated by the CFD simulations, a BPNN was implemented in Python through the utilisation of Keras. This network enables the real-time prediction to be achieved. The structure and key parameters of this BPNN are determined through cross-verification, as illustrated in Figure 10. The inputs of BPNN consist of parameters such as the accelerating voltage, beam current, weld speed, and the beam radii (1/e^2^ widths) in both the x and y directions. The output represents the penetrated depth value. The machine learning model was trained using data generated through the CFD method.

Firstly, the parameters that could potentially impact BPNN prediction performance were identified. The activation functions, which determine how neurons are activated based on equations, such as Softplus equation *f*(*x*) = *log*(*exp*(*x*) + 1), were considered, as different activation functions can affect the prediction accuracy and efficiency. In addition to the activation function, the number of hidden layers, neurons number of each layer, learning rate, decay rate, and loss type were considered in this study.

Secondly, cross-verification was performed to establish the structure of the BPNN, utilising simulation data divided into four equal sets. Three sets were utilised as training data to predict the remaining set, and this process was repeated four times by rotating the test data sets. The cross-verification method aims to identify the appropriate neural network structure and parameters while mitigating the risk of overfitting. The activation function, the number of hidden layers, the number of neurons in each layer, the learning rate, decay rate, and loss type can be determined when the prediction deviation reaches its minimum during cross-verification.

Finally, the model established using paired data generated by CFD simulations was then validated using actual experimental data.

## 5. Results and Discussion

**Figure 11:**
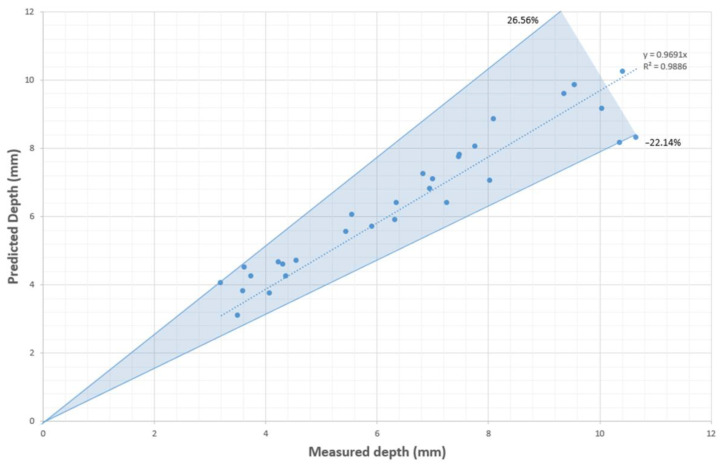
Simulated depths via CFD modelling compared with measured depths of C1–C30.

The simulated results of the depth were compared with the experimentally measured depth of C1–C30, as shown in Table 4 and Figure 11. It is important to note that in some cases, the penetration depth of a single-path weld is not consistent, and may even decrease over time. This inconsistency can be attributed to the potential collapse of the keyhole due to gravitational effects. Hence, it was necessary to record five depths at different times. The simulated average absolute percentage deviation was found to be 8.26%, and the maximum absolute percentage deviation was 26.56%. Taking into account the accumulated error (typically around 4%) caused by variances in machine input, the measurement method from microscope images, measurements at different weld positions, and fluctuations in substrate temperature, the overall deviation of approximately 8% demonstrates that the CFD model is an efficient method for predicting the electron beam weld penetration depth before conducting the weld.

If we set a requirement of less than 15% deviation, 26 out of 30 predictions fall within this range. To be more specific, the predictions with significant deviations are listed in Table 5.

When compared to ANN with experimental data from previous studies whose average absolute percentage deviations went as low as 5% [2], the prediction accuracy of the CFD model is notably lower. Table 5 shows that significant errors occur only in over-focus situations with an accelerating voltage of 40 kV and under-focus situations with 60 kV. For the welds with a 40 kV accelerating voltage, the large deviations are all positive, indicating that the CFD method overestimates the penetration depth of these welds. This is likely due to the lower kinetic energy of single electrons at 40 kV, making them more susceptible to ambient particle effects. As a result, the efficiency of 40 kV welds may be lower than that of 50 kV and 60 kV welds, a feature not reflected in the current CFD model. On the other hand, for the welds with an accelerating voltage of 60 kV, the large deviations are all negative, further supporting the above statement.

Additionally, the under-focus status, which can generate deeper penetration during EBW compared with the over-focus situations [35,36], is not appropriately reflected in the current model, thus amplifying the errors in Table 5. However, there is potential to enhance the CFD model by incorporating the focus status of the electron beam, a topic that will be explored in future studies.

Despite not reaching the same level of prediction accuracy as the ANN trained with adequate actual data, the CFD model still shows promise as it reduces the cost and time of conducting experiments. It took approximately 30 h to complete the simulation for each model, and multiple models can run simultaneously without affecting each other. However, the ANN model relies on extensive trial-and-error testing, which typically spans a few weeks. According to the data in [2], when the number of training experiments is below 14, the CFD model performs better. Thus, it can be concluded that CFD models can offer superior prediction accuracy compared to BPNNs trained with limited experimental data.

**Figure 12:**
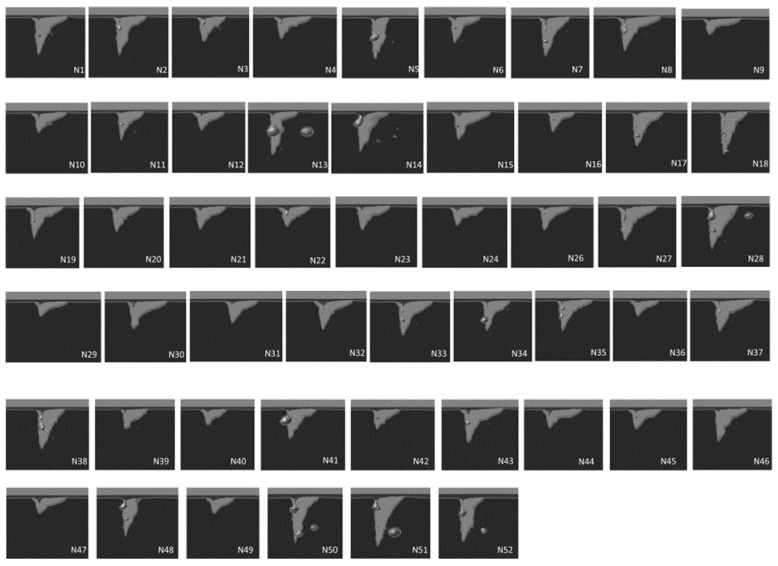
Sectional view of the molten pools simulated by the CFD model (N1–N52).

Based on the established CFD data, the fast-responding ANN model is able to be built based on CFD-generated paired data. When configuring the model, it is essential to define the range of welding parameters, as a specific welding scenario must be provided for making predictions. However, it is not feasible to enumerate all of the potential parameter combinations within that range. Therefore, orthogonal experimental design proves to be an effective method for addressing this issue. Table 6 displays the CFD-simulated penetration depth using the weld parameters designed via the orthogonal experimental design approach using the commercial software SPSS (version 29), including beam current (25 mA, 30 mA, 35 mA, 40 mA, and 45 mA), accelerating voltage (40 kV, 50 kV, and 60 kV), welding speed (500 mm/min, 550 mm/min, 600 mm/min, 650 mm/min, and 700 mm/min), and beam 1/e^2^ radii in both the x and y directions (0.25 mm, 0.35 mm, 0.45 mm, 0.55 mm, 0.65 mm, and 0.75 mm). Tests C1–C30 listed in Table 4 also fall within the range of these parameters. Figure 12 illustrates the sectional view of the molten pools in the 52 simulation cases, where the light area represents the liquid phase, and the dark area represents the solid phase.

Utilising the 52 CFD-simulated data from Table 6, the BPNN model can be trained and the structure confirmed as follows:

(1) One hidden layer with 15 neurons.
(2) Linear transfer function for the input layer and ‘softplus’ activation function for the hidden layer.
(3) Type ‘Normal’ kernel initialiser for the input layer and the hidden layer.
(4) ‘SGD’ optimiser: Initial learning rate is 0.001, decay steps 10,000, and decay rate 0.9.
(5) Losses type: mean absolute percentage error.
(6) 5000 iterations.

**Figure 13:**
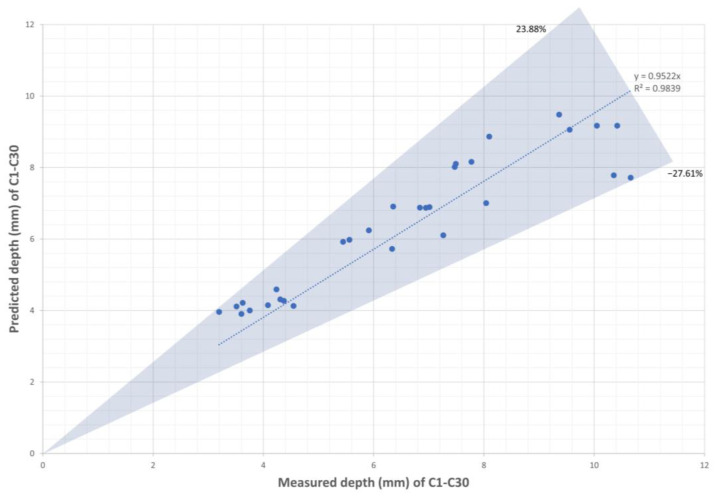
Predicted depths by CFD-based BPNN compared with measured depths of C1–C30.

The predictions made by the BPNN were subsequently compared with the actual depths in Table 1. The results are presented in Figure 13.

With 52 training data points, the BPNN achieved a prediction with an average absolute percentage deviation of 9.19%. The prediction deviations range from +23.88% to −27.61%. If we set a requirement of less than 15% deviation, 24 out of 30 predictions fall within this range. It can be observed that the prediction accuracy of the BPNN is slightly lower than that of the CFD model. Although it may be possible to improve the BPNN accuracy to closer to that of the CFD prediction by increasing the quantity of paired data or adjusting the neural network structure, surpassing the CFD prediction seems unlikely, as the generated data are already affected by the lack of focal position information.

The ML-enhanced CFD model significantly reduced the calculation time to less than 1 s compared to the 30 h required by the CFD model alone. Remarkably, this efficiency improvement has not led to a significant compromise in the prediction accuracy. The results confirm the feasibility and efficiency of the ML-enhanced CFD model.

## 6. Conclusions

In this study, a novel CFD approach was employed to predict the penetration depth during electron beam welding. The CFD model yields satisfactory prediction results, with an average absolute percentage deviation of approximately 8%. The advantage of this approach lies in its ability to reduce costs and save time, since it does not require extensive testing before building the model, unlike statistical methods. The prediction method can also be adapted to different materials by modifying the CFD material properties, and to different machines by following the beam characterisation method.

Furthermore, in this study, virtual paired data between weld parameters and penetration depth were generated using the CFD model, allowing the tuning of neural networks for real-time and fast response prediction in real industrial scenarios. The ML-enhanced CFD model demonstrates the ability to achieve rapid predictions, exhibiting an average absolute percentage deviation of roughly 9%, without the need for expensive trial-and-error experimentation.

However, the current ML-enhanced CFD model does have some limitations. The model does not yet incorporate detected beam focal position information, leading to certain prediction deviations. Additionally, errors may arise when the electron beam power density deviates from the ideal Gaussian distribution. The current beam probing method falls short in accurately describing non-Gaussian beams. These challenges persistently affect the development of EBW automation and prediction transfer among different machines. The next objective is to enhance the simulation accuracy of the CFD model by incorporating additional information, such as the focal position and the impact index of the accelerating voltage. Additionally, the aim is to improve the beam probing method by integrating more detection directions and implementing automatic height adjustments. These enhancements will empower the ML-enhanced CFD model to offer more accurate and improved depth prediction results.

## Figures and Tables

**Table 1:** Welding parameters and measured penetration depths for each trial [2].

Weld No.	Accelerating Voltage *U* (kV)	Beam Current *I* (mA)	Welding Speed *S* (mm/min)	Focusing Current and Relative Sharp Focus Current *I_fr_* (mA)	Beam Radius (1/e^2^ Width *σ_x_*) at x Direction (mm)	Beam Radius (1/e^2^ width *σ_y_*) at y Direction (mm)	Measured Penetration Depth ± Standard Deviation (mm)
C1	40	45	650	280 (sharp-focus −6)	0.571	0.704	4.24 ± 0.09
C2	40	30	700	293 (sharp-focus +8)	0.413	0.467	3.20 ± 0.05
C3	50	40	700	316 (sharp-focus −4)	0.377	0.455	6.36 ± 0.09
C4	50	30	550	320 (sharp-focus −4)	0.266	0.277	6.95 ± 0.19
C5	60	45	500	340 (sharp-focus −8)	0.520	0.517	10.66 ± 0.17
C6	60	40	500	362 (sharp-focus +4)	0.339	0.369	9.55 ± 0.26
C7	60	40	700	354 (sharp-focus −4)	0.330	0.346	9.36 ± 0.27
C8	60	35	500	351 (sharp-focus −4)	0.261	0.304	10.42 ± 0.38
C9	60	30	650	360 (sharp-focus)	0.208	0.277	8.10 ± 0.18
C10	50	35	500	312 (sharp-focus −8)	0.407	0.439	6.33 ± 0.20
C11	50	25	550	329 (sharp-focus +4)	0.251	0.319	5.56 ± 0.10
C12	40	45	700	294 (sharp-focus +8)	0.778	0.901	3.51 ± 0.10
C13	40	40	650	278 (sharp-focus −8)	0.501	0.622	4.31 ± 0.08
C14	40	25	500	293 (sharp-focus +4)	0.333	0.400	3.62 ± 0.11
C15	40	35	600	285 (sharp-focus)	0.435	0.540	4.55 ± 0.07
C16	60	40	600	366 (sharp-focus +8)	0.401	0.423	7.77 ± 0.16
C17	60	25	500	353 (sharp-focus −8)	0.312	0.266	8.04 ± 0.26
C18	60	35	550	351 (sharp-focus −4)	0.261	0.304	10.05 ± 0.35
C19	40	45	650	286 (sharp-focus)	0.631	0.794	4.37 ± 0.05
C20	40	45	650	290 (sharp-focus +4)	0.702	0.829	4.08 ± 0.04
C21	40	35	650	277 (sharp-focus −8)	0.450	0.571	3.75 ± 0.05
C22	40	30	600	293 (sharp-focus +8)	0.413	0.467	3.60 ± 0.07
C23	50	30	700	316 (sharp-focus −8)	0.336	0.350	5.45 ± 0.13
C24	50	25	650	321 (sharp-focus −4)	0.246	0.274	5.91 ± 0.44
C25	50	35	600	320 (sharp-focus)	0.304	0.366	7.01 ± 0.25
C26	50	40	550	324 (sharp-focus +4)	0.370	0.434	6.84 ± 0.31
C27	50	45	500	326 (sharp-focus +8)	0.495	0.605	7.26 ± 0.25
C28	60	25	650	357 (sharp-focus −4)	0.222	0.233	7.47 ± 0.23
C29	60	30	550	364 (sharp-focus +4)	0.250	0.322	7.49 ± 0.20
C30	60	45	600	340 (sharp-focus −8)	0.520	0.517	10.36 ± 0.23

**Table 2:** Materials properties for S275JR applied in CFD model [30,31,32].

Physical Property	Value
Thermal conductivity	52.11 W/(m × K) at 300 K
Density	7840 kg/m³
Latent heat of fusion	288,482 J/kg
Solidus temperature	1743 K
Liquidus temperature	1788 K
Specific heat	830 J/(kg × K) at 300 K
Surface tension	1.8 N/m at 1850 K
Boiling point	3135 K
Viscosity	0.003 kg/(m × s) at 1800 K
Molecular	55.845 kg/kmol

**Table 3:** Some settings of the Fluent CFD model for simulating S275JR bead-on-plate EBW.

Model Parameters	Setting Value/Introduction
Mutiphase model	Homogenous model, Volume of Fluid (VOF)
Interface type	Sharp
Phases	Two phases (gas phase as the main phase)
Phase interaction	Continuum surface force with wall adhesion
Mushy zone parameter	4,000,000
Pressure–velocity coupling	SIMPLE
Solution controls	Default

**Table 4:** CFD-simulated results of the depth compared with experimentally measured depths of samples C1–C30.

No.	Depth 1 (mm)	Depth 2 (mm)	Depth 3 (mm)	Depth 4 (mm)	Depth 5 (mm)	Average Simulated Depth (mm)	Actual Depth (mm)
C1	4.75	4.5	4.75	4.5	4.75	4.65	4.24
C2	4.25	3.75	4.25	3.75	4.25	4.05	3.2
C3	6.25	6.75	6.25	6.5	6.25	6.4	6.36
C4	6.5	7	6.75	7	6.75	6.8	6.95
C5	8.25	8.5	8.25	8.25	8.25	8.3	10.66
C6	9.75	10	9.75	10	9.75	9.85	9.55
C7	9	10	9.75	9.75	9.5	9.6	9.36
C8	10.25	10.25	10.25	10.25	10.25	10.25	10.42
C9	8.75	8.75	9	8.75	9	8.85	8.1
C10	5.5	6.5	6	6	5.5	5.9	6.33
C11	6	6.25	6	6	6	6.05	5.56
C12	3	3.5	3	3	3	3.1	3.51
C13	4.25	4.75	4.75	4.75	4.5	4.6	4.31
C14	4.25	4.25	5	4.25	4.75	4.5	3.62
C15	4.5	4.5	4.75	5	4.75	4.7	4.55
C16	7.75	8.25	8	8.25	8	8.05	7.77
C17	6.5	7.25	7.25	7.25	7	7.05	8.04
C18	9	9.25	9.25	9.25	9	9.15	10.05
C19	3.75	4.5	4.25	4.5	4.25	4.25	4.37
C20	3.75	3.75	3.75	3.75	3.75	3.75	4.08
C21	4.25	4.25	4.25	4.25	4.25	4.25	3.75
C22	3.75	4	3.75	3.75	3.75	3.8	3.6
C23	5.5	5.75	5.25	5.75	5.5	5.55	5.45
C24	5.5	6	5.75	5.75	5.5	5.7	5.91
C25	7	7	7.25	7.25	7	7.1	7.01
C26	6.75	7.5	7.5	7.5	7	7.25	6.84
C27	6.25	6.5	6.5	6.5	6.25	6.4	7.26
C28	7.5	8	7.75	7.75	7.75	7.75	7.47
C29	7.5	8.5	7.5	8	7.5	7.8	7.49
C30	8	8.75	8	8	8	8.15	10.36

**Table 5:** List of cases with absolute deviation of penetration depth predictions larger than 15%.

	Accelerating Voltage (kV)	Beam Current *I* (mA)	Welding Speed *S* (mm/min)	Focal Position	Deviation of CFD Prediction
C2	40	30	700	Over-focus (+8 mA)	+26.56%
C5	60	45	500	Under-focus (−8 mA)	−22.14%
C14	40	25	500	Over-focus (sharp-focus +4)	+24.31%
C30	60	45	600	Under-focus (−8 mA)	−21.33%

**Table 6:** CFD-simulated penetration depths of N1–N52.

No.	Accelerating Voltage *U* (kV)	Beam Current *I* (mA)	Welding Speed *S* (mm/min)	Beam Radius (1/e^2^ Width *σ_x_*) at x Direction (mm)	Beam Radius (1/e^2^ Width *σ_y_*) at y Direction (mm)	Depth at 1.2 s (mm)	Depth at 1.4 s (mm)	Depth at 1.6 s (mm)	Depth at 1.8 s (mm)	Depth at 2 s (mm)	Average Depth (mm)
N1	50	40	600	0.25	0.55	7.25	7.5	7.25	7.25	7.25	7.3
N2	40	45	550	0.35	0.35	6.75	7.5	7.5	7.5	7	7.25
N3	60	30	550	0.75	0.25	4.75	5.5	5.25	5.5	4.75	5.15
N4	60	25	700	0.65	0.45	3.75	3.75	4.25	4	3.75	3.9
N5	60	35	500	0.35	0.25	8	8.25	8.25	8.25	8	8.15
N6	50	25	600	0.45	0.35	4.75	4.75	5	5	4.75	4.85
N7	60	40	500	0.55	0.35	7.5	7.75	7.5	7.75	7.5	7.6
N8	60	40	700	0.45	0.65	6.5	7	6.75	6.75	6.5	6.7
N9	40	30	700	0.35	0.75	3	3	3	3.5	3	3.1
N10	40	35	550	0.45	0.55	3.75	4.5	3.75	4.25	3.75	4
N11	40	25	500	0.25	0.25	5.25	5.5	5.5	5.5	5.5	5.45
N12	50	30	500	0.65	0.55	3.5	3.75	3.75	3.75	3.75	3.7
N13	40	45	700	0.25	0.25	8	8.25	8.25	8.5	8.25	8.25
N14	50	30	700	0.25	0.25	7.5	7.5	7.5	7.5	7.5	7.5
N15	50	35	700	0.75	0.35	5	6	5.25	6	5.25	5.5
N16	40	25	600	0.35	0.45	3.75	4.25	3.75	3.75	3.75	3.85
N17	60	30	550	0.45	0.25	6	6.75	6.5	6.75	6.25	6.45
N18	60	35	550	0.25	0.45	8.25	8.5	8.25	8.5	8.25	8.35
N19	60	30	500	0.35	0.55	6	6	6	6	6	6
N20	50	30	550	0.55	0.25	4.75	5	5	5	4.75	4.9
N21	50	25	500	0.65	0.25	3.75	4.25	4.25	4.25	3.75	4.05
N22	40	40	500	0.75	0.45	3.75	4.25	4.25	4.25	3.75	4.05
N23	40	35	600	0.55	0.25	3.75	4.5	4.5	4.5	4.5	4.35
N24	40	30	500	0.55	0.65	3	3.5	3	3.5	3	3.2
N25	50	25	550	0.35	0.65	4.25	4.75	4.5	4.75	4.5	4.55
N26	60	45	650	0.75	0.55	6.5	6.75	6.5	6.5	6.5	6.55
N27	40	40	550	0.65	0.75	3.5	3.75	3.75	3.75	3.75	3.7
N28	50	40	650	0.35	0.25	8.25	8.25	8.5	8.5	8.25	8.35
N29	40	25	700	0.55	0.55	3	2.75	3	3	3	2.95
N30	60	25	550	0.25	0.75	4.5	5.25	5	5.25	4.75	4.95
N31	40	30	500	0.45	0.45	4.25	4.25	4.25	4.25	4.25	4.25
N32	50	45	500	0.45	0.75	5.5	6	5.75	5.75	5.75	5.75
N33	50	45	550	0.55	0.45	6.5	7	6.5	7	6.5	6.7
N34	60	25	500	0.25	0.35	5.75	6.75	6	6	5.75	6.05
N35	60	30	600	0.25	0.65	6	6	6	6	6	6
N36	40	30	600	0.75	0.75	2.75	3	3	3	3	2.95
N37	50	35	500	0.25	0.75	5.25	5.5	5.5	5.75	5.5	5.5
N38	60	45	600	0.65	0.25	7.75	8.5	8.25	8.25	8	8.15
N39	40	25	550	0.25	0.55	3.75	4.25	3.75	3.75	3.75	3.85
N40	40	25	500	0.75	0.25	3	3	3	3	3	3
N41	40	30	650	0.25	0.35	5.25	5.75	5.5	5.75	5.5	5.55
N42	40	25	650	0.45	0.25	3.75	3.75	3.75	3.75	3.75	3.75
N43	40	45	500	0.25	0.65	5.5	6.5	6.25	6.25	6	6.1
N44	60	25	650	0.55	0.75	3.5	3.75	3.5	3.5	3.5	3.55
N45	40	30	550	0.65	0.35	3.75	3.75	3.75	3.75	3.75	3.75
N46	50	30	650	0.25	0.45	6.25	5.75	6.25	6.25	6.25	6.15
N47	40	35	650	0.65	0.65	3	3.75	3.5	3.5	3.5	3.45
N48	40	40	550	0.25	0.25	6.5	7.5	7	7.25	6.75	7
N49	50	25	550	0.75	0.65	3	3	3	3	3	3
N50	50	45	500	0.25	0.25	10	10.5	10.5	10.5	10.5	10.4
N51	50	40	550	0.25	0.35	8	9.5	9.25	9.5	8.75	9
N52	50	40	600	0.35	0.25	8.5	8.5	8.5	8.5	8.5	8.5

## Data Availability

Data underlying this study can be accessed through the Cranfield University repository at https://doi.org/10.17862/cranfield.rd.24427033 (accessed on 11 September 2023).
